# Effect of antipsychotic on mismatch negativity amplitude and evoked theta power in drug-naïve patients with schizophrenia

**DOI:** 10.1186/s12888-024-06314-w

**Published:** 2024-12-18

**Authors:** Yan-Bing Xiong, Qi-Jing Bo, Xian-Bin Li, Yi Liu, Qi-Bo Guo, Chuan-Yue Wang

**Affiliations:** 1 Department of Psychiatry, Tongji Shanxi Hospital, Shanxi Bethune Hospital, Shanxi Academy of Medical Sciences, Third Hospital of Shanxi Medical University, Taiyuan, China; 2 Tongji Hospital, Tongji Medical College, Huazhong University of Science and Technology, Wuhan, China; 3 The National Clinical Research Center for Mental Disorders & Beijing Key Laboratory of Mental Disorders & Beijing Institute for Brain Disorders Center of Schizophrenia, Beijing Anding Hospital, Capital Medical University, Beijing, China; 4 Advanced Innovation Center for Human Brain Protection, Capital Medical University, Beijing, China

**Keywords:** Schizophrenia, Mismatch negativity, Time-frequency analysis, Antipsychotic

## Abstract

**Background:**

Recurrent observations have indicated the presence of deficits in mismatch negativity (MMN) among schizophrenia. There is evidence suggesting a correlation between increased dopaminergic activity and reduced MMN amplitude, but there is no consensus on whether antipsychotic medications can improve MMN deficit in schizophrenia.

**Methods:**

We conducted clinical assessments, cognitive function tests, and EEG data collection and analysis on 31 drug-naïve patients with schizophrenia. Comprehensive evaluation tools such as PANSS and MCCB. MMN amplitude was analyzed by event-related potential (ERP) approaches, evoked theta power was analyzed by event-related spectral perturbation (ERSP) approaches.

**Results:**

Our findings indicate that antipsychotic treatment significantly improved clinical symptoms, as evidenced by reductions in PANSS positive, negative, general symptoms, and total scores (all *p* < 0.001). Cognitive function improvements were observed in language learning, working memory, and overall MCCB scores (*p* < 0.05), although other cognitive domains showed no significant changes. However, no significant improvements were noted in MMN amplitude and evoke theta power after four weeks of antipsychotic treatment (*p* > 0.05).

**Conclusion:**

These results suggest that while antipsychotic medications effectively alleviate clinical symptoms, their impact on MMN amplitude and evoke theta power deficit is limited in the short term. Moreover, the amelioration of cognitive impairment in individuals with schizophrenia is not readily discernible, and it cannot be discounted that the enhancement observed in language acquisition and working memory may be attributed to a learning effect. These findings underscore the complexity of the neurobiological mechanisms involved and highlight the need for further research to optimize individualized treatment strategies for schizophrenia.

**Trial Registration:**

ChiCTR2000038961, October 10, 2020.

**Supplementary Information:**

The online version contains supplementary material available at 10.1186/s12888-024-06314-w.

## Introduction

Schizophrenia is a severe mental disorder that significantly impacts patients’ cognitive functions, social capabilities, and overall quality of life. With a prevalence rate of approximately 1%, schizophrenia imposes substantial psychological and economic burdens on patients and society [1]. More than 50% of those observed experience intermittent yet persistent psychiatric problems, while approximately 20% suffer from lifelong chronic clinical symptoms and disability. This not only causes severe harm to the individuals but also imposes a significant burden on society, with the anticipated disease burden expected to more than double by 2030 [2]. Therefore, reducing the increasing disease burden of mental disorders such as schizophrenia has become a priority in mental health and is also important for improving the social functioning of patients. However, the current prevention, control, and diagnosis of schizophrenia are not ideal mainly due to difficulties in early identification and intervention in patients with schizophrenia, resulting in poor prognosis for these individuals and an inability to effectively restore their social functions [3].

Auditory mismatch negativity (MMN) is an event-related potential (ERP) that occurs when an auditory stimulus with different properties, such as duration (dMMN) or frequency (fMMN), is presented together with a standard stimulus that is more frequently presented [4]. MMN serves as an indicator of a memory-driven cerebral auditory sensory reaction to identifiable alterations in a sequence of stimuli, even when attention is not present [5]. The MMN component of the evoked potential has been discovered to offer valuable insights into the importance of regularity sensitivity in perception and cognition [6]. The MMN paradigm has gained considerable attention in recent years due to its capacity to provide insights into the underlying mechanisms of sensory information processing by detecting deviations [7, 8]. The correlation between MMN and both neurocognitive abilities and functional outcomes among individuals diagnosed with schizophrenia has been well-established in prior research [9, 10].

Cognitive impairment constitutes a fundamental manifestation of schizophrenia [11]. Previous research has consistently demonstrated that individuals with schizophrenia exhibit cognitive deficits across multiple domains, consistently performing 0.5–1.75 standard deviations below the mean of healthy individuals on neuropsychological tasks assessing various cognitive functions [12]. The recovery of cognitive function plays a pivotal role in determining the reintegration of individuals with schizophrenia into society. Biomarkers hold great potential for the early diagnosis of schizophrenia, and among various neurophysiological and neurocognitive measures, MMN reduction stands out as one of the most robust findings in patients with this disorder. Our previous study revealed a significant association between MMN amplitude impairment in individuals with schizophrenia and deficits in word learning and working memory within the cognitive domain [13].

Previous study has demonstrated the lack of efficacy of antipsychotic medication in ameliorating MMN deficits among individuals diagnosed with schizophrenia [14]. However, some studies have also found that antipsychotic medication improves MMN impairment in schizophrenia [15]. The cause of the variability in these results is unknown, and further follow-up studies are required. Furthermore, previous follow-up studies investigating antipsychotic therapy for MMN deficiency in patients with schizophrenia have primarily focused on assessing the amplitude of MMN. Relative to MMN amplitude, which can only provide local potential information on evoked potentials, event related spectral perturbation (ERSP) analysis can provide both potential-level and molecular-level information, in addition to more complex physiological information [16]. It has been shown that the ERSP evoked power of auditory MMN is mainly concentrated in the theta band (4–7 Hz), and the presence of impaired ERSP energy in the theta band evoked by MMN was found to be a biomarker of schizophrenia [17]. Our group also found that impaired evoked theta power deficits in schizophrenia was associated with working memory [13]. However, there are no current studies on follow-up studies of antipsychotic drugs inducing theta power deficits in patients with schizophrenia.

In summary, both MMN amplitude and evoked theta power can serve as potential biomarkers for precise interventions targeting cognitive impairment in schizophrenia; however, the effect of antipsychotics on MMN wave amplitude remains inconclusive based on previous studies, and there is a lack of follow-up research investigating the impact of antipsychotic treatment on evoked theta power. In this study, we administered a 4-week course of antipsychotic treatment to drug-naïve patients with schizophrenia in order to investigate the impact of antipsychotics on both MMN amplitude and evoked theta power, aiming to elucidate whether antipsychotics can effectively ameliorate these deficits. The findings from this research have the potential to inform the development of more efficacious therapeutic strategies for managing cognitive deficits in schizophrenia, ultimately enhancing patient outcomes.

## Method and material

### Participants

Thirty-one drug-naïve individuals diagnosed with schizophrenia were recruited as participants from Beijing Anding Hospital, Capital Medical University. The inclusion criteria for this study involved the validation of diagnoses using the Structured Clinical Interview for DSM-IV (SCID). Participants ranged in age from 18 to 45 years and all had IQ scores equal to or greater than 70. Exclusion criteria encompassed hearing impairments, learning difficulties, neurological disorders, a history of seizures or head injuries, prior electroconvulsive therapy, and substance abuse. This study obtained approval from the Ethics Committee of Beijing Anding Hospital and all subjects provided informed consent before participating.

### Procedures

The auditory stimuli comprised a series of binaural tones (825 trials) presented in a random sequence with a stimulus onset asynchrony ranging from 500 to 550 ms. The majority of the trials (675, accounting for 82%) featured standard tones characterized by a frequency of 1000 Hz, sound intensity level of 75 dB, and duration of 50 ms. In contrast, deviant tones included variations in both frequency and duration. Frequency deviants (75 trials, representing 9% of the total) had a frequency of 1500 Hz, sound intensity level of 75 dB, and duration of 50 ms. Duration deviants (another set comprising approximately 9% or 75 trials overall) maintained the same frequency as the standard tones but had an extended duration lasting for 100 ms instead. To establish baseline standards at the experiment’s outset, we employed the initial set consisting of fifteen stimuli as our reference.

### Electroencephalogram (EEG) data acquisition and processing

Electroencephalogram (EEG) data were recorded from all participants using a 128-channel electrode system (Electrical Geodesics, Inc., Oregon, USA) with standard reference and grounding procedures. The signal impedance was adjusted to be ≥ 50 KΩ while maintaining a sampling rate of 1000 Hz. During the experiment, subjects were seated comfortably in a specially designed room that minimized potential external factors that could affect the study. The test consisted of three sections separated by breaks lasting for 60 s each.

For the ERP analyses, we employed EEGLAB 14.1.1b (http://sccn.ucsd.edu/eeglab/), a MATLAB-based tool for neural electrophysiological analysis. The EEG data were processed using a finite impulse response filter with a bandpass range of 0.1–40 Hz to ensure optimal signal quality. To eliminate power frequency noise at 50 Hz, notch processing was applied as an additional step in the preprocessing pipeline. We adopted a global brain average reference as the new electrode configuration to enhance spatial comparability across subjects and minimize potential bias introduced by individual differences in scalp topography. Independent component analysis (ICA) was utilized to effectively remove artifacts caused by eye movement, ensuring accurate interpretation of the underlying neural activity patterns. Segmentation of the EEG data encompassed a time window from 100 ms before stimulus onset to 500 ms after stimulus initiation, allowing us to capture both pre-stimulus baseline activity and post-stimulus responses within an appropriate temporal context for further analysis purposes. MMN waveforms were derived by subtracting the deviant stimulus from the standard stimulus specifically at the frontal midline (Fz) electrode location.

For the analysis of evoked (average) power, we employed the short-time Fourier transformation (STFT) method in MATLAB (MathWorks, Natick, MA, USA) to convert ERP waves. The segmented EEG signal underwent continuous wavelet transformation over time. The EEG data spanned from 100 ms prior to stimulus initiation to 500 ms after stimulus onset relative to presentation time. A frequency range of 1–20 Hz was applied for the wavelet transformation. Furthermore, temporal power values corresponding to each frequency point were averaged across trials to obtain an EEG power time-frequency distribution on a channel-by-channel basis. For statistical analysis purposes, maximum power values within each subject’s theta frequency band of 4–7 Hz and between 100 and 250 ms were extracted. This range represents the primary active frequency band of neural oscillation in response to the standard stimulus.

### Clinical, intelligence quotient and neuropsychological assessment

The clinical symptoms of each patient were evaluated with the Positive and Negative Symptom Scale (PANSS, Chinese version), which was previously described [18]. The Chinese intelligence quotient (IQ) test tool was revised short form the Wechsler adult intelligence scale-revised, and the four included subsets for this evaluation were information, similarities, picture completion, and block design [19]. The MATRICS consensus cognitive battery (MCCB, Chinese version) was used to evaluated cognitive deficits in patients with schizophrenia and healthy controls [20].

### Statistical analysis

The statistical analysis was performed using SPSS 20.0 (IBM, Chicago, IL, USA). To compare PANSS scores, MCCB scores, amplitudes of MMN and evoke power between the baseline and 4-week treatment sessions, we utilized paired-samples T tests. A significance level of *p* < 0.05 was employed for the statistical analysis.

## Results

### Demographics and clinical characteristics

The mean age of individuals diagnosed with schizophrenia was 27.5 ± 5.7 years, with a majority (77%) being male. The average duration of education for these individuals was 13.9 ± 3.5 years, while the mean age at onset of symptoms was recorded as 25.6 ± 5.3 years, and the average illness duration stood at 26.4 ± 30.7 months. Furthermore, the conversion to olanzapine-equivalent dosage resulted in an average daily intake of 15.9 ± 6.3 mg/dose per patient’s requirement. The detailed results are shown in Table 1. The medication information for each patient is provided in Table S1.

**Table 1:** General demographic and clinical data characteristics

Variables	Patients (*N* = 31)
Age (mean ± SD, year)	27.5 ± 5.7
Sex (male / %)	24 / 77%
Education years (mean ± SD, year)	13.9 ± 3.5
Onset age (mean ± SD, year)	25.6 ± 5.3
Course (mean ± SD, month)	26.4 ± 30.7
olanzapine-equivalent dosage (mean ± SD, mg/d)	15.9 ± 6.3

### Comparisons of PANSS before and after antipsychotics treatment

After a 4-week treatment regimen with a single antipsychotic medication, there was a significant improvement in the symptoms of patients diagnosed with schizophrenia. Specifically, statistically significant differences were observed in PANSS positive symptom scores (df = 30, t = 11.991, *p* < 0.001), negative symptom scores (df = 30, t = 8.650, *p* < 0.001), general symptom scores (df = 30, t = 14.086, *p* < 0.001), and the overall PANSS score (df = 30, t = 16.898 *p* < 0.001). The detailed results are shown in Table 2.

**Table 2:** PANSS scores before and after antipsychotic treatment

Variables	Baseline		After 4-week treatments		Paired-samples T tests		
	Mean	SD	Mean	SD	df	t	*p* (2-tailed)
Positive symptom scale	21.2	4.1	10.7	2.4	30	11.991	< 0.001
Negative symptom scale	17.9	5.3	11.7	3.1	30	8.650	< 0.001
Total psychopathology scale	38.7	4.3	25.4	3.1	30	14.086	< 0.001
PANSS (total scores)	77.8	9.5	47.5	6.6	30	16.898	< 0.001

### Comparisons of MCCB before and after antipsychotics treatment

The neurocognitive treatment using MCCB demonstrated significant improvements in word learning (df = 30, t = -3.393, *p* = 0.002), working memory (df = 30, t = -3.766, *p* = 0.001), and total MCCB cognitive score (df = 30, t = -3.042, *p* = 0.005). However, no significant improvements were observed in other cognitive domains. Specific detailed results are shown in Table 3.

**Table 3:** MCCB scores before and after antipsychotic treatment

Variables	Baseline		After 4-week treatments		Paired-samples T tests		
	Mean	SD	Mean	SD	df	t	*p* (2-tailed)
Speed of processing	36.9	6.7	38.6	7.3	30	-1.349	0.188
Attention/Vigilance	34.1	8.7	34.5	11.5	30	-0.238	0.814
Working memory	39.7	8.9	45.2	7.1	30	-3.766	**0.001**
Verbal learning	37.5	9.3	42.1	9.6	30	-3.393	**0.002**
Visual learning	44.3	11.2	48.2	11.8	30	-1.829	0.077
Reasoning and problem solving	39.4	12.1	42.7	10.9	30	-1.864	0.072
Social cognition	32.9	7.9	33.6	10.0	30	-0.389	0.700
MCCB combine	37.7	5.7	40.7	6.1	30	-3.042	**0.005**

### Comparisons of MMN index and after antipsychotics treatment

**Fig. 1:**
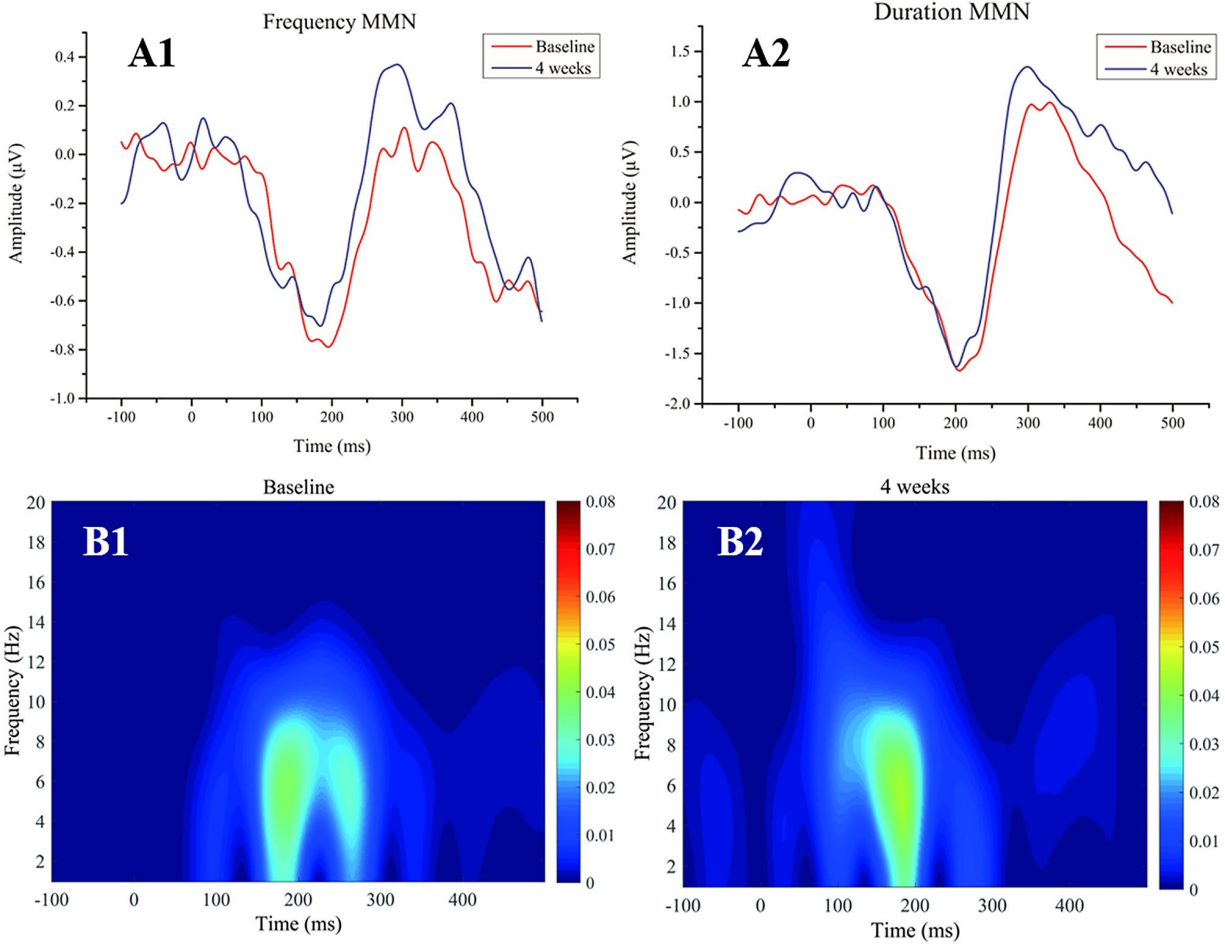
(**A1**-**A2**) Mismatch negativity amplitudes before and after antipsychotic treatment. (**B1**-**B2**) evoke power before and after antipsychotic treatment

After 4 weeks of monotherapy with antipsychotic medication, there was no statistically significant improvement observed in the amplitude of frequency MMN (df = 30, t = -1.043, *p* = 0.305), the amplitude of duration MMN (df = 30, t = -0.403, *p* = 0.690), and evoke theta power (df = 30, t = -1.242, *p* = 0.224). Detailed results are shown in Table 4; Fig. 1.

**Table 4:** Mismatch negativity amplitudes and evoke power before and after antipsychotic treatment

Variables	Baseline		After 4-week treatments		Paired-samples T tests		
	Mean	SD	Mean	SD	df	t	*p* (2-tail)
Frequency MMN (µV)	-0.7911	0.2884	-0.7443	0.2951	30	-1.043	0.305
Duration MMN (µV)	-1.6588	0.5394	-1.6212	0.5370	30	-0.403	0.690
Evoke theta power	0.0381	0.0338	0.0427	0.0364	30	-1.242	0.224

## Discussion

This study focuses on the neurocognitive performance and auditory evoked potentials, specifically MMN amplitude and its related frequency band analysis, in drug-naïve patients with schizophrenia before and after antipsychotic treatment. Previous studies investigating the effects of antipsychotic drugs on MMN indexes in schizophrenia have predominantly focused on amplitude, whereas this study represents the first attempt to examine the influence of antipsychotic drugs on MMN evoked power in individuals with schizophrenia. Notably, our findings reveal that while antipsychotic treatment significantly improves clinical symptoms, its impact on cognitive function and neurophysiological markers such as MMN amplitude and theta evoke power is limited.

Cognitive dysfunction in patients with schizophrenia is considered a significant pathological manifestation, persisting even after treatment of core clinical symptoms such as hallucinations and delusions. Moreover, it closely correlates with the functional outcome of patients. The process of cognitive change over the course of schizophrenia remains controversial. The current general research view is that the cognitive function of patients with schizophrenia is basically stable over time [21, 22], but some studies have found that cognitive function declines over time and even improves in some cognitive areas [23, 24]. When comparing the individual cognitive function between patients with first-episode and chronic schizophrenia, no statistically significant differences were observed in the function of each cognitive domain [13]. This finding supports the notion that as the disease progresses, individuals with schizophrenia maintain a stable state in terms of their cognitive function. Furthermore, several studies have reported impaired cognitive function in clinical high risk (CHR) [25, 26] and among first-degree relatives (FDRs) [27, 28], suggesting that cognitive deficits in schizophrenia patients could serve as an indicator of vulnerability. Interestingly, this study revealed that non-medicated individuals with schizophrenia who received antipsychotic treatment for a duration of 4 weeks exhibited enhancements in word learning and working memory. In order to elucidate this finding, we initially considered the potential influence of a short-term follow-up period on the observed practice effect. However, Jahshan C [23] conducted a 6-month follow-up study on first-episode schizophrenia and observed that speech learning exhibited the most significant enhancement. Consequently, it can be inferred with accuracy that while the majority of cognitive functions in individuals with schizophrenia remain stable throughout the course of the disease, specific cognitive abilities such as word learning may exhibit improvement through interventions like medication.

Impairment of MMN amplitude in schizophrenia is considered to be one of the most potentially robust biomarkers for schizophrenia [29]. Previous studies have demonstrated a more pronounced impairment of MMN amplitude in individuals with chronic schizophrenia compared to those with first-episode schizophrenia; however, no significant association has been observed between the extent of MMN amplitude impairment and the progression of schizophrenia [30]. One potential explanation is that the decline in MMN amplitude among individuals with schizophrenia progressively deteriorates within 1–2 years following diagnosis, and subsequently stabilizes after reaching a critical stage. This hypothesis is supported by a study demonstrating a correlation between the impairment of MMN amplitude and disease progression during the initial 18 months [31]. The findings of this study revealed a decrement in MMN amplitude as the 4-week disease course progressed. Although no significant statistical difference was observed, these results may suggest that the observed decline in MMN amplitude among individuals with schizophrenia follows a non-linear growth trajectory, indicating an absence of linear correlation [30]. Furthermore, our results suggest that the decline in MMN amplitude may worsen as the disease progresses, potentially indicating a progressive nature of MMN deficits due to cortical tissue loss in areas associated with attention regulation and orienting [32] or medication-related effects.

The findings from our trial indicate that antipsychotics do not effectively ameliorate MMN amplitude and impairment in patients with schizophrenia. One possible explanation for the previous conflicting results on whether antipsychotics improve MMN amplitude is that drugs with a strong serotonergic influence, such as aripiprazole [33] or quetiapine [15] tablets, may enhance MMN amplitude. A separate study demonstrated that the administration of escitalopram tablets to healthy participants resulted in a significant modulation of MMN amplitude. This observation suggests that the regulation of MMN may involve serotonergic mechanisms [34]. However, MMN deficits are not improved by other second-generation antipsychotics that have a strong affinity for serotonin receptors [35]. In addition, our findings indicate that antipsychotics do not show any significant impact on the enhancement of evoke theta power.

Numerous hypotheses have been proposed to explain the mechanisms underlying MMN impairment in individuals with schizophrenia; however, the most widely acknowledged hypothesis among researchers is that it is associated with deficient NMDAR function in this patient population. The impaired function of NMDAR in patients with schizophrenia is widely acknowledged within the field of schizophrenia research. Previous investigations have demonstrated a decline in mRNA expression levels and diminished protein levels specifically of N1-type NMDA receptors within the prefrontal cortex among individuals diagnosed with this disorder [36]. Both studies conducted on non-human primates using intracranial [37] and surface [38] recording techniques, as well as investigations involving healthy volunteers [39] administered with ketamine (an NMDAR antagonist), have provided evidence suggesting that insufficient activity of individual NMDR receptors may be associated with MMN impairment. Importantly, this impairment is not believed to have any correlation with 5-hydroxytryptamine (5-HT) receptors or dopamine receptor function [40]. Recent studies have found that D-serine (which enhances NMDA receptor function as an endogenous ligand at the NMDAR regulatory site [41] or glycine (which enhances NMDAR function) [42] ameliorates MMN impairment in schizophrenic patients, corroborating that MMN impairment in schizophrenic patients may be related to their NMDA receptor insufficiency. As well as, it is preferable that antipsychotics do not ameliorate MMN impairment as the impairment of evoke theta power is also linked to NMDAR and can be enhanced by N-methyl-D-aspartic acid receptor (NMDAR) modulators [43].

## Limitation

Despite the strengths of our study, several limitations must be acknowledged. The sample size of 31 patients is relatively small, which may limit the generalizability of our findings. The four-week follow-up period is also relatively short, preventing us from assessing the long-term effects of antipsychotic treatment on cognitive and neurophysiological outcomes. Future research should aim to include larger, more diverse samples and extend the follow-up period to capture long-term effects. The ERP paradigm does not control for stimulus-specific effects, which means it utilizes the deviant as a standard in an alternating recording to calculate MMN as difference waves from the alternate recording (using a ‘flip/flop design’), and future studies need to refine the MMN paradigm. Furthermore, combining antipsychotic treatment with cognitive behavioral therapy (CBT) or other psychosocial interventions and recording global function levels (GAF) could provide a more comprehensive understanding of how to address the multifaceted nature of schizophrenia.

## Conclusion

In conclusion, our study highlights the efficacy of antipsychotic medications in improving clinical symptoms of schizophrenia but also underscores the need for additional interventions to address cognitive deficits and MMN abnormalities. These findings have important implications for clinical practice and policy-making, suggesting that a more integrated treatment approach may be necessary to fully support the recovery of schizophrenia patients. Future research should focus on larger, more diverse populations and longer follow-up periods to validate and extend these findings.

## Electronic supplementary material

Below is the link to the electronic supplementary material.

Supplementary Material 1

## Data Availability

Data is provided within the manuscript or the corresponding author, CYW, upon reasonable request.
