# Supplementary material for: Effect of antipsychotic on mismatch negativity amplitude and evoked theta power in drug-naïve patients with schizophrenia
Source: BMC Psychiatry. 2024 Dec 18;24:901. doi: 10.1186/s12888-024-06314-w (PMC11653912; doi:10.1186/s12888-024-06314-w)
Supplement: Supplementary file 1 — Supplementary Material 1 [file 12888_2024_6314_MOESM1_ESM.docx]

**Table S1** Medication information for each patient

| **Number** | **Therapeutic drug** | **Dosage (mg/d)** | **olanzapine-equivalent dosage (mg/d)** | **drug delivery route** |
| --- | --- | --- | --- | --- |
| 1 | olanzapine | 20 | 20 | oral |
| 2 | aripiprazole | 15 | 10 | oral |
| 3 | risperidone | 5 | 10 | oral |
| 4 | amisulpride | 1000 | 25 | oral |
| 5 | aripiprazole | 30 | 20 | oral |
| 6 | olanzapine | 20 | 20 | oral |
| 7 | aripiprazole | 20 | 13.3 | oral |
| 8 | olanzapine | 20 | 20 | oral |
| 9 | olanzapine | 15 | 15 | oral |
| 10 | amisulpride | 1000 | 25 | oral |
| 11 | olanzapine | 20 | 20 | oral |
| 12 | risperidone | 8 | 16 | oral |
| 13 | risperidone | 6 | 12 | oral |
| 14 | risperidone | 4 | 8 | oral |
| 15 | aripiprazole | 20 | 13.3 | oral |
| 16 | olanzapine | 20 | 20 | oral |
| 17 | amisulpride | 1200 | 30 | oral |
| 18 | risperidone | 4 | 8 | oral |
| 19 | amisulpride | 1000 | 25 | oral |
| 20 | aripiprazole | 20 | 13.3 | oral |
| 21 | olanzapine | 15 | 15 | oral |
| 22 | aripiprazole | 10 | 6.7 | oral |
| 23 | paliperidone | 12 | 20 | oral |
| 24 | olanzapine | 20 | 20 | oral |
| 25 | amisulpride | 300 | 7.5 | oral |
| 26 | amisulpride | 400 | 10 | oral |
| 27 | risperidone | 6 | 12 | oral |
| 28 | ziprasidone | 40 | 5 | oral |
| 29 | paliperidone | 12 | 20 | oral |
| 30 | paliperidone | 12 | 20 | oral |
| 31 | risperidone | 6 | 12 | oral |
